# In Vivo Assessment of the Calcium Salt-Forming Ability of a New Calcium Silicate-Based Intracanal Medicament: Bio-C Temp

**DOI:** 10.3390/dj11040091

**Published:** 2023-03-30

**Authors:** Naoki Edanami, Razi Saifullah Ibn Belal, Shoji Takenaka, Kunihiko Yoshiba, Rosa Edith Baldeon Gutierrez, Shintaro Takahara, Nagako Yoshiba, Naoto Ohkura, Yuichiro Noiri

**Affiliations:** 1 Division of Cariology, Operative Dentistry and Endodontics, Department of Oral Health Science, Graduate School of Medical and Dental Sciences, Niigata University, Niigata 951-8514, Japan; 2 Division of Oral Science for Health Promotion, Department of Oral Health and Welfare, Graduate School of Medical and Dental Sciences, Niigata University, Niigata 951-8514, Japan

**Keywords:** apexification, calcite, calcium silicate-based intracanal medicament, hydroxyapatite, rat subcutaneous implantation

## Abstract

Calcium salt precipitation induced by intracanal medicaments contributes to the formation of apical hard tissue during apexification. This study compared the calcium salt-forming ability of a new calcium silicate-based intracanal medicament (Bio-C Temp) with that of two commercial calcium hydroxide pastes (Calcipex Plane II and Vitapex) in a rat subcutaneous implantation model. Polytetrafluoroethylene tubes containing each of the three materials were subcutaneously implanted in 4-week-old male Wistar rats. After 28 days, the composition and amount of calcium salts formed at the material–tissue interface were assessed using micro-Raman spectroscopy, X-ray diffraction, and elemental mapping. The tested materials produced white precipitates that had Raman spectra with peaks corresponding to hydroxyapatite and calcite. X-ray diffraction detected hydroxyapatite formation on Calcipex Plane II and Vitapex implants, as well as calcite formation on all three materials. Elemental mapping revealed that Bio-C Temp generated significantly smaller calcium- and phosphorus-rich calcified regions within the subcutaneous connective tissue than Vitapex. These results indicate that Bio-C Temp produced less calcium salt in rat subcutaneous tissue than Vitapex, although all materials formed hydroxyapatite and calcite in rat subcutaneous tissue. Bio-C Temp could be less effective than Vitapex in promoting apical hard tissue formation during apexification.

## 1. Introduction

The aim of apexification is to promote apical hard tissue formation for root-end closure in immature pulpless teeth. This can be achieved through the application of an intracanal dressing of calcium hydroxide paste or through a root canal filling with calcium silicate cement [1]. There are advantages to both types of treatments. Apexification using calcium silicate cement requires fewer treatment sessions and shorter treatment durations than that using calcium hydroxide paste. Apexification using calcium hydroxide paste costs less, and retreatment is easier than when using calcium silicate cement. Moreover, a meta-analysis reported that the clinical success rates of the two procedures were comparable [2].

Recently, a ready-to-use calcium silicate paste called Bio-C Temp (Angelus, Londrina, PR, Brazil) was developed and marketed. Bio-C Temp is a unique calcium silicate-based material that does not harden in root canals as other conventional calcium silicate-based endodontic materials do. The manufacturer claims that Bio-C Temp can be used as an intracanal dressing in apexification as an alternative to calcium hydroxide pastes [3]. However, it has not been fully investigated whether Bio-C Temp possesses appropriate physicochemical and biological properties as an agent for apexification [3,4,5,6,7].

An intracanal dressing with calcium hydroxide paste induces calcium salt precipitation in the apical connective tissue, and the calcium salts contribute to apical hard tissue formation during apexification [8]. When calcium hydroxide pastes interact with periapical connective tissue, the release of hydroxyl ions (OH^−^) from the pastes induces the formation of a superficial necrotic layer in the connective tissue [8]. Subsequently, calcium carbonate and calcium phosphate crystals precipitate adjacent to the necrotic layer due to interactions between calcium ions (Ca^2+^) and carbonate or phosphate ions derived from the materials and tissue fluids, respectively [9,10,11]. The calcium salts bind to various bioactive molecules including fibronectin [10,12,13], osteopontin [14], and dentin matrix protein-1 [15], thereby rendering the surfaces for attachment and differentiation of hard tissue-forming cells [8,9,10,11,12,13].

Bio-C Temp releases Ca^2+^ and OH^−^ via hydration [5]. Therefore, Bio-C Temp may also induce calcium salt precipitation in adjacent connective tissue through the above reactions. Although an assessment of the calcium salt-forming ability of Bio-C Temp may be useful in predicting its therapeutic effects in apexification, this ability has not yet been reported.

The ability of materials to induce calcium salt precipitation in vivo is often assessed by implanting the materials into rat subcutaneous connective tissue [16,17]. This method is optimal because subcutaneous connective tissue does not produce biological hard tissues (such as dentin, cementum, and bone) that hinder calcium salt detection.

Therefore, in this study, we evaluated the calcium salt-forming ability of Bio-C Temp in comparison with that of two commercial calcium hydroxide pastes, namely, Calcipex Plane II (Nishika, Shimonoseki, Japan) and Vitapex (Neo Dental Chemical Products, Tokyo, Japan) using the rat subcutaneous implantation model. The null hypothesis of this study was that Bio-C Temp and the two commercial calcium hydroxide pastes do not exhibit differences in calcium salt formation in rat subcutaneous tissue.

## 2. Materials and Methods

### 2.1. Ethical Approval

All animal experiments were approved by the Committee on the Guidelines for Animal Experimentation of Niigata University (approval number: SA00912) and conducted according to all applicable international, national, and/or institutional guidelines for the care and use of animals.

### 2.2. Materials

Bio-C Temp, Calcipex Plane II, and Vitapex were assessed. Table 1 shows the composition of these materials.

### 2.3. Rat Subcutaneous Implantation

In total, 11 four-week-old male Wistar rats weighing 70–80 g were purchased from Clea Japan (Tokyo, Japan). The animals were housed in standardized conditions at a temperature of 23 °C ± 2 °C and a humidity of 40–70% under a 12 h light/dark cycle with ad libitum access to water and a commercial pellet diet.

The rats were anesthetized with an intraperitoneal injection of medetomidine hydrochloride, midazolam, and butorphanol. After dorsal skin shaving, three 5 mm long incisions were made on the back of each animal using No. 11 scalpel blades (Feather, Osaka, Japan). The incisions were laterally extended and surgical pockets were created. Closed-end polytetrafluoroethylene (PTFE) tubes (length: 5 mm; outer diameter: 3 mm; and inner diameter: 2 mm) loaded with Bio-C Temp, Calcipex Plane II, or Vitapex were then inserted into the surgical pockets, and the incisions were sutured with 4–0 silk (Mani, Tochigi, Japan). After the surgery, the animals were observed daily by veterinary technicians. No alterations in behavior were noted throughout the experimental period. The animals were euthanized by anesthetic overdose 28 days after implantation, and the implants were collected along with the surrounding tissue. The specimens were divided into two groups: one group (*n* = 6 per material) was used for stereomicroscopic, micro-Raman spectrometric, and X-ray diffraction (XRD) analyses to examine the appearance and composition of precipitates formed on the implants. The other group (*n* = 5 per material) was used for elemental mapping to compare the degree of calcium salt formation by each material. The sample size for the former group (*n* = 6 per material) was determined based on our preliminary data to obtain a sufficient amount of precipitate for the micro-Raman and XRD analyses (>5 mm^3^). The sample size for the latter group (*n* = 5 per material) was calculated considering an effect size of 1, power of 0.8, and α-value of 0.05 using G*Power software (University of Dusseldorf, Dusseldorf, Germany).

### 2.4. Stereomicroscopy

The retrieved tissue specimens containing the implants (*n* = 6 per material) were immersed in 6% sodium hypochlorite for 20 min to remove the connective tissue from the implants. Precipitate formation on the material surface was examined using a stereomicroscope (SMZ800; Nikon, Tokyo, Japan) equipped with a digital camera (DSFi2; Nikon).

### 2.5. Micro-Raman Spectrometry

The samples used in Section 2.4. (*n* = 6 per material) were subsequently used for micro-Raman spectrometry. Precipitates formed on the implants were collected by gentle scraping using a dental excavator. The six precipitate samples for each material were combined together, ground using an agate mortar, and analyzed using a Raman spectrometer connected to a microscope at 100× magnification (NRS-3100; JASCO, Tokyo, Japan). Six spectra were recorded for each material. The Raman spectra were acquired using a 3 s integration time with 7 co-addition scans. A laser beam with an excitation wavelength of 532 nm was used. The laser power was set to 7.4 mW. Band assignments were performed as previously described [18,19,20].

### 2.6. XRD

The precipitates collected from the implants in Section 2.5. were further analyzed using XRD (Ultima IV; Rigaku, Tokyo, Japan). The 2θ range of 25°–40° was analyzed with a step size of 0.02°. The scan rate was 1° per 2.5 min. The copper X-ray source was operated at 40 kV and 40 mA. The assignments of the diffraction peaks were performed using the Crystallography Open Database (http://www.crystallography.net, accessed on 1 February 2023).

### 2.7. Elemental Mapping 

The degree of calcium salt formation by each material was evaluated using elemental mapping of the material–tissue interfaces. The retrieved specimens (*n* = 5 per material) were fixed in 2.5% glutaraldehyde solution buffered with 60 mmol/L HEPES for 24 h at 4 °C, dehydrated with increasing ethanol concentrations and acetone, and embedded in methacrylate resin (Osteoresin; Wako, Osaka, Japan). The resin-embedded specimens were longitudinally sectioned through the center of the implants using a water-cooled diamond wheel saw (MC-201N; Maruto, Tokyo, Japan), polished with silicon-carbide paper (4000 grit), gold-coated with an ion coater (IC-50; Shimadzu, Kyoto, Japan), and analyzed using an electron probe microanalyzer (EPMA1601; Shimadzu) to map calcium (Ca) and phosphorus (P) at the material–tissue interface. The area size of the elemental mapping was 2.27 mm × 2.27 mm, the step size was 15 μm × 15 μm, the sampling time was 0.03 s at each point, and the accelerating voltage was 15 kV.

The total area of the Ca- and P-rich regions within the subcutaneous connective tissue was calculated using ImageJ software 1.53t (National Institutes of Health, Bethesda, MD, USA). 

### 2.8. Statistical Analysis 

The area of the Ca- and P-rich regions produced by each material was compared. Data were statistically analyzed using one-way analysis of variance, followed by Tukey’s post hoc test. SPSS 10 software for Windows (SPSS Japan, Tokyo, Japan) was used for the analysis. The level of statistical significance was set at 5%.

## 3. Results

### 3.1. Stereomicroscopy

**Figure 1:**
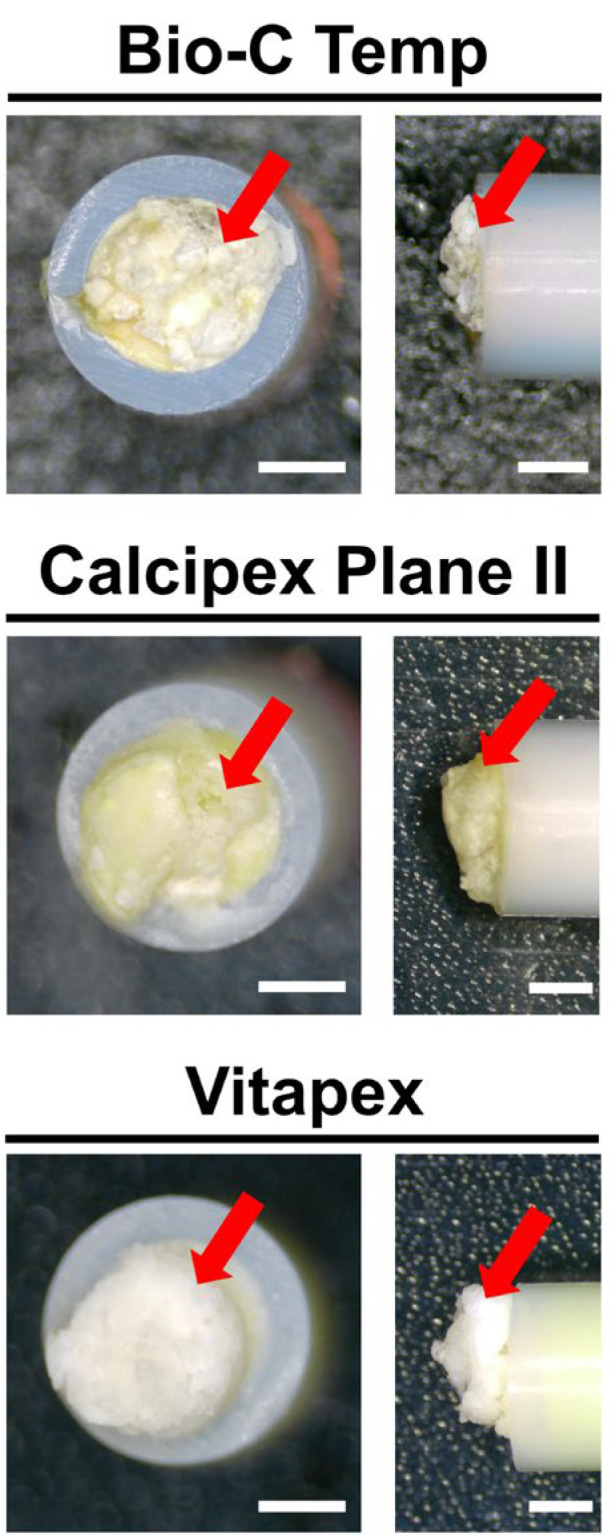
Stereomicroscopic images of the Bio-C Temp, Calcipex Plane II, and Vitapex implants retrieved from rat subcutaneous tissue 28 days after implantation. White precipitates can be observed on the tube openings (arrows). Scale = 1 mm.

White precipitates with an irregular shape were observed on all implanted materials. The surfaces of all Vitapex implants and one Calcipex Plane II implant were completely covered by precipitates, whereas those of the other implants were not. The images are shown in Figure 1.

### 3.2. Micro-Raman Spectrometry

**Figure 2:**
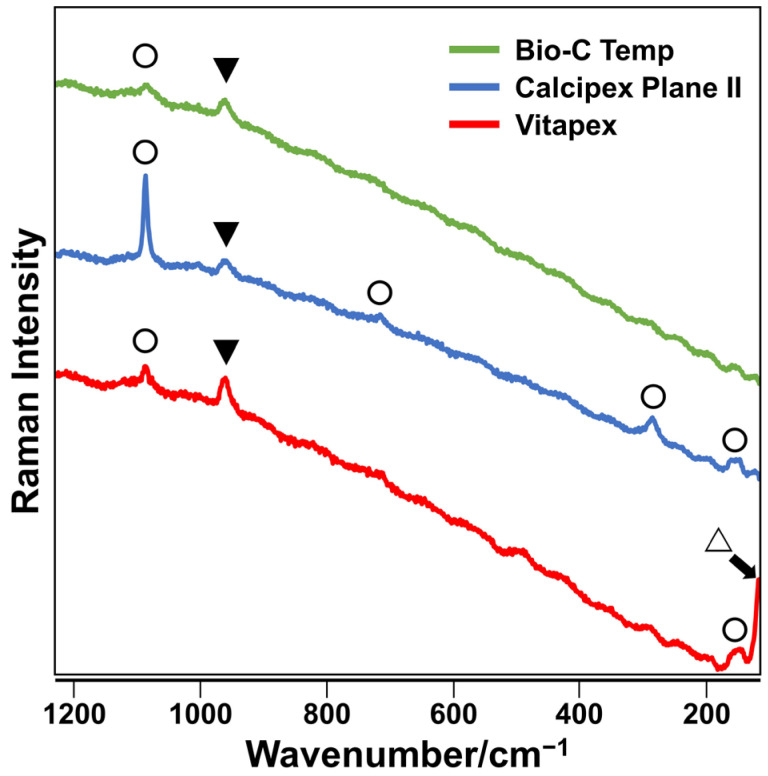
Representative Raman spectra of precipitates formed on the Bio-C Temp, Calcipex Plane II, and Vitapex implants. Bands corresponding to hydroxyapatite (▼), calcite (◯), and iodoform (△) are shown.

The precipitates formed on the Bio-C Temp and Calcipex Plane II implants exhibited Raman spectra peaks corresponding to hydroxyapatite and calcite, whereas those formed on Vitapex exhibited Raman spectra peaks corresponding to hydroxyapatite, calcite, and iodoform (Figure 2).

### 3.3. XRD

**Figure 3:**
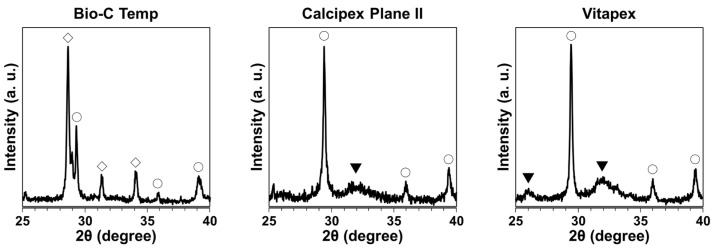
XRD patterns of the precipitates formed on the Bio-C Temp, Calcipex Plane II, and Vitapex implants. ▼: hydroxyapatite, ◯: calcite, ◇: calcium tungstate.

The XRD pattern for the precipitates on the Bio-C Temp implants corresponded to calcite and calcium tungstate peaks, whereas the XRD pattern for those on the Calcipex Plane II and Vitapex implants corresponded to calcite and hydroxyapatite peaks (Figure 3).

### 3.4. Elemental Mapping

**Figure 4:**
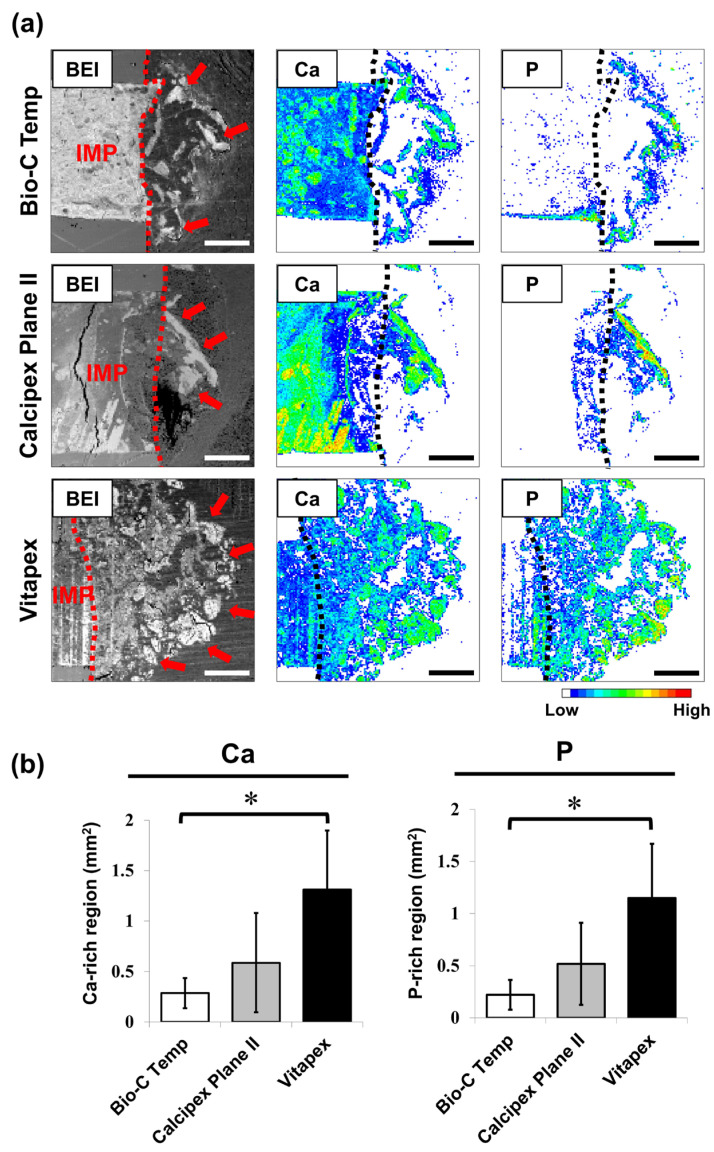
Degree of calcium salt formation around the Bio-C Temp, Calcipex Plane II, and Vitapex implants. (**a**) Representative backscattered electron images (BEI) and elemental mapping images of the subcutaneous tissue–implant interfaces. Dotted lines indicate the surface of the implants. Arrows indicate the calcium (Ca)- and phosphorus (P)-rich calcified regions in the subcutaneous tissue. (**b**) Average area sizes of the Ca-rich and P-rich regions within the subcutaneous tissue. Data are presented as means ± SD (Tukey’s test, *n* = 5, * *p* < 0.05). Scale = 500 μm. IMP: implant.

The results of the elemental mapping analysis are presented in Figure 4. Ca- and P-rich calcified regions were observed in the subcutaneous tissue adjacent to the implanted materials (Figure 4a). The Bio-C Temp specimens exhibited significantly smaller Ca-rich and P-rich regions in the subcutaneous tissue than the Vitapex specimens (Figure 4b; *p* < 0.05, Tukey’s test). No significant differences were detected for the other comparisons.

## 4. Discussion

This study demonstrated that Bio-C Temp produced two distinct forms of calcium salts, namely, hydroxyapatite and calcite, in rat subcutaneous tissue, similar to Calcipex Plane II and Vitapex. However, Bio-C Temp produced less calcium salt than Vitapex in rat subcutaneous tissue. Thus, the null hypothesis of this study that the calcium salt formation in rat subcutaneous tissue does not differ between the three materials was rejected.

Calcium hydroxide pastes are categorized into the following three types: those containing aqueous vehicles, viscous vehicles, and oily vehicles [21]. In this study, an aqueous vehicle-containing calcium hydroxide paste (Calcipex Plane II) and an oily vehicle-containing calcium hydroxide paste (Vitapex) were used as comparisons against Bio-C Temp. Aqueous vehicle-containing calcium hydroxide pastes are more soluble and dissolve faster in the adjacent tissue than oily vehicle-containing calcium hydroxide pastes [21]. Moreover, a previous study reported that an oily vehicle-containing calcium hydroxide paste allowed for a faster formation of apical hard tissue during apexification than an aqueous vehicle-containing calcium hydroxide paste [22].

In this study, the materials were implanted in rat subcutaneous tissue for 28 days to provide adequate time for calcium salt precipitation. Calcium salt precipitation starts within 7 days after subcutaneous implantation of calcium hydroxide-releasing materials in rats [17,23]. Additionally, PTFE tubes were used as molds for the subcutaneous implantation of the tested materials in this study. The subcutaneous implantation of PTFE tubes alone for 28 days does not produce a detectable amount of calcium salts in rat tissue [23].

Previous studies used the von Kossa birefringent method to detect calcium salt precipitation in connective tissue [24,25]. However, a detailed composition of the precipitates cannot be determined using this method. In addition, some precipitates might be lost during the thin sectioning and staining steps used in this method. Therefore, in the current study, we performed micro-Raman spectroscopy and XRD to determine the composition of the precipitates. Moreover, we evaluated the degree of calcium salt precipitation in the subcutaneous connective tissue using elemental mapping, which does not involve thin sectioning and histological staining. 

After the subcutaneous implantation in rats, all the tested materials produced white precipitates (Figure 1). Micro-Raman analysis revealed that the precipitates included hydroxyapatite and calcite (Figure 2). XRD confirmed the presence of hydroxyapatite in the precipitates on Calcipex Plane II and Vitapex, as well as the presence of calcite in the precipitates on all three materials (Figure 3). Although hydroxyapatite was not detected using XRD in the Bio-C Temp precipitates (Figure 3), this could be because the amount of hydroxyapatite produced by Bio-C Temp was too small to be detected via XRD. A previous study reported that the micro-Raman technique was more sensitive than the XRD technique for detecting hydroxyapatite [26].

In the micro-Raman and XRD analyses, iodoform and calcium tungstate were detected in the Vitapex precipitates and the Bio-C Temp precipitates, respectively (Figure 2 and Figure 3). However, these components seemed to be contaminants from the substrate materials, because Vitapex contains iodoform and Bio-C Temp contains calcium tungstate (Table 1). 

While this is the first study to evaluate the calcium salt-forming ability of Bio-C Temp, previous studies have demonstrated that various formulations of calcium silicate-based endodontic materials form calcium salts, including hydroxyapatite and calcite, when in contact with artificial or real body fluids [27,28]. The current study revealed that calcium silicate-based Bio-C Temp produced hydroxyapatite and calcite in rat subcutaneous tissue. The results of the current study are in agreement with those of previous studies.

In the elemental mapping analysis, the hydroxyapatite and calcite deposits were observed as Ca-rich and P-rich regions in the subcutaneous tissue adjacent to the implanted materials (Figure 4a). As shown in Figure 4b, both the Ca-rich and P-rich regions produced by Bio-C Temp were significantly smaller than those produced by Vitapex. This finding indicates that Bio-C Temp produced less calcium salt (hydroxyapatite and calcite) in rat subcutaneous tissue than Vitapex.

The lower amount of calcium salts produced by Bio-C Temp as compared with Vitapex may be mainly attributable to the differences in their Ca^2+^ and OH^−^ release and cytotoxicity. Bio-C Temp has lower Ca^2+^ and OH^−^ releasing abilities [5] and higher cytotoxicity [3,4] than calcium hydroxide pastes. An environment rich in Ca^2+^ and OH^−^ is favorable for calcium salt precipitation [29]. In addition, inflammation caused by cytotoxic materials produces an acidic environment, which is unfavorable for calcium salt precipitation [29,30].

Moreover, the presence of silicone oil in Vitapex might also be responsible for the greater amount of calcium salt formation on Vitapex than on Bio-C Temp. A previous study reported that alkali-treated silicone gums produced calcium salts in an artificial body fluid [31]. Thus, the silicone oil in Vitapex might facilitate calcium salt formation under the alkaline environment created by the calcium hydroxide components of Vitapex.

A previous study showed that Bio-C Temp released Ca^2+^ and OH^−^ similar to calcium hydroxide pastes [5]. Ca^2+^ and OH^−^ stimulate the osteo/cementogenic differentiation of periapical cells [32,33]. Moreover, this study showed that Bio-C Temp and the two types of calcium hydroxide pastes shared the ability to produce hydroxyapatite and calcite in vivo. Hydroxyapatite and calcite act as scaffolds for bone and cementum matrix deposition [34,35]. Based on these findings, it can be assumed that Bio-C Temp promotes apical hard tissue formation during apexification through a mechanism similar to that of calcium hydroxide pastes. However, in this study, Bio-C Temp formed less calcium salt in rat subcutaneous tissue than Vitapex. In previous studies, a resin-free calcium silicate cement (ProRoot MTA) produced more calcium salts in rat subcutaneous tissue [23] and induced faster reparative dentin formation in rat molars than a resin-modified calcium silicate cement (TheraCal LC) [36]. Moreover, biomaterials that produced more calcium salts in an artificial body fluid formed more ectopic bone in dog muscle [37]. Thus, Bio-C Temp, which produced less calcium salt in rat subcutaneous tissue than Vitapex, could be less effective than Vitapex in promoting apical hard tissue formation during apexification, although further confirmation is necessary. 

A limitation of this study was the difference between the experimental environment and clinical settings. Dentin substances, which were absent in this study, could influence calcium salt precipitation by neutralizing the alkaline environment produced by intracanal medicaments [38]. Additionally, non-collagenous proteins in apical connective tissue promote or inhibit calcium salt precipitation [39]. In future studies, the calcium salt-forming ability of Bio-C Temp should be evaluated under conditions that resemble clinical settings, closely using root canal treatment models in rats [40,41] or dogs [42].

Overall, Bio-C Temp had a weaker ability to produce hydroxyapatite and calcite in rat subcutaneous tissue than Vitapex. This suggests that the effects of Bio-C Temp on the apical hard tissue formation during apexification are less potent than those of Vitapex. Further studies are required to clarify the therapeutic effect of Bio-C Temp.

## Figures and Tables

**Table 1:** Composition of the materials used in this study.

Material	Manufacturer	Composition
Bio-C Temp	Angelus, Londrina, PR, Brazil	Tricalcium silicate, dicalcium silicate, tricalcium aluminate, calcium oxide, base resin, calcium tungstate, polyethylene glycol, and titanium oxide
Calcipex Plane II	Nishika, Shimonoseki, Japan	Calcium hydroxide (48%), purified water, and others
Vitapex	Neo Dental Chemical Products, Tokyo, Japan	Iodoform (40.4%), calcium hydroxide (30.3%), silicone oil (22.4%), and others

## Data Availability

The datasets generated and/or analyzed during the current study are available from the corresponding author upon reasonable request.
